# Fabrication Process Research for Silicon-Waveguide-Integrated Cavity Optomechanical Devices Using Magnesium Fluoride Protection

**DOI:** 10.3390/mi16111217

**Published:** 2025-10-26

**Authors:** Chengwei Xian, Pengju Kuang, Ning Fu, Zhe Li, Changsong Wang, Yi Zhang, Rudi Zhou, Guangjun Wen, Boyu Fan, Yongjun Huang

**Affiliations:** 1 School of Information and Communication Engineering, Sichuan Provincial Engineering Research Center of Communication Technology for Intelligent IoT, University of Electronic Science and Technology of China, Chengdu 611731, China; 2 School of Electronic Engineering, Chengdu Technological University, Chengdu 611730, China; 3 School of Automation Engineering, University of Electronic Science and Technology of China, Chengdu 611731, China

**Keywords:** integrated silicon waveguide, magnesium fluoride, hydrofluoric acid etching, selective protection, SOI micromachining

## Abstract

As an emerging platform for high-precision sensing, integrated silicon-waveguide-based cavity optomechanical devices face a critical fabrication challenge in the co-fabrication of silicon-on-insulator (SOI) micromechanical structures and optical waveguides: the silicon oxide (SiO_2_) layer beneath the waveguides is susceptible to etching during hydrofluoric acid (HF) release of the microstructures, leading to waveguide collapse and significantly reducing production yields. This study proposes a novel selective protection process based on a magnesium fluoride (MgF_2_) thin film to address the critical challenge of long-range waveguide collapse during hydrofluoric acid (HF) etching. By depositing a MgF_2_ protective layer over the waveguide regions via optical coating technology, localized protection of specific SiO_2_ areas during HF etching is achieved. The experimental results demonstrate the successful release of silicon waveguides with lengths of up to 5000 μm and a significant improvement in production yield. This work provides a compatible and efficient strategy for the fabrication of robust photonic–microelectromechanical integrated devices.

## 1. Introduction

The silicon-on-insulator (SOI) platform has emerged as an ideal substrate for realizing high-performance, miniaturized integrated optical sensors—such as accelerometers—due to its outstanding optical confinement, mature complementary metal–oxide–semiconductor (CMOS)-compatible fabrication processes, and inherent compatibility with microelectromechanical systems (MEMSs) [1,2,3,4]. In silicon-based integrated optical sensors, optical waveguides generally serve as the core sensing elements, whose structural integrity—particularly in suspended sections—is critical to the device’s sensitivity, stability, and reliability [5,6]. Either wet or vapor hydrofluoric acid (HF) etching is a standard process for releasing micromechanical structures—such as proof masses, cantilevers, and waveguides—from the SOI device layer, primarily by selectively removing the buried oxide (BOX) layer to achieve structural suspension [7,8].

However, a significant technical challenge arises during the release of microstructures that incorporate optical waveguides: as HF laterally undercuts the BOX layer beneath the waveguides, capillary forces during the release process and Van der Waals forces after release readily cause the suspended waveguide structures to collapse or adhere (stiction) to the underlying silicon substrate [9,10]. This structural failure not only disrupts the optical transmission path—resulting in severe degradation of optical performance (e.g., transmission loss)—but also significantly compromises the mechanical sensing functionality and overall device yield of the sensor. To mitigate this issue, several alternative approaches have been explored. For instance, supercritical CO_2_ drying can be employed as a remedial measure post wet etching [11], or vapor HF etching can be utilized to entirely avoid liquid-phase processes [12]. However, the former only addresses capillary forces during the drying stage and remains costly, while the latter, though free from capillary effects, still suffers from isotropic etching behavior that leads to lateral undercutting of the SiO_2_ beneath waveguides, failing to ensure the integrity of long waveguide structures.

To address the aforementioned challenges, this work proposes and demonstrates a novel, efficient solution that is compatible with standard micro- and nanofabrication processes: a magnesium fluoride (MgF_2_) layer is introduced as a selective protective coating over the regions to be preserved beneath the waveguides prior to the vapor HF release step. The core idea of this strategy is to leverage the exceptional chemical inertness and high corrosion resistance of MgF_2_ against gaseous HF [13,14]. In stark contrast to silicon dioxide (SiO_2_) BOX layers, which are readily etched by HF, MgF_2_ exhibits an extremely low etch rate in HF vapor. This critical property ensures that the BOX regions accurately covered by the MgF_2_ film remain completely intact during the HF release process, thereby physically blocking lateral undercutting by HF in these protected zones. As a result, this mechanism fundamentally eliminates the possibility of the supporting structures under the waveguides being hollowed out, effectively avoiding the risk of post-release collapse due to insufficient anchor support. Furthermore, MgF_2_ possesses good optical transparency in the near-infrared communication band (e.g., 1550 nm) [15,16], making its potential influence on optical transmission controllable.

Based on this approach, we detail a microfabrication process for SOI waveguide-based sensors that utilizes an MgF_2_ protective layer to achieve high resistance to HF corrosion. This paper systematically elucidates the operating principles and design considerations of the proposed protection scheme; comprehensively describes the development of a complete process flow, including MgF_2_ deposition, vapor HF release, and MgF_2_ removal; and provides strong experimental validation through rigorous characterization involving scanning electron microscopy (SEM) of critical structures and optical pathway testing. The results demonstrate the remarkable effectiveness of the MgF_2_ layer in preventing HF corrosion of the buried oxide (BOX) beneath the waveguides and suppressing their collapse. This study offers a reliable and scalable fabrication strategy with significant potential for industrial adoption in manufacturing high-integrity, high-performance integrated optoelectronic MEMS sensors.

## 2. Principle and Design

### 2.1. Chip Architecture and Operating Principle of the Integrated Silicon Waveguide Cavity Optomechanical Sensor

**Figure 1:**
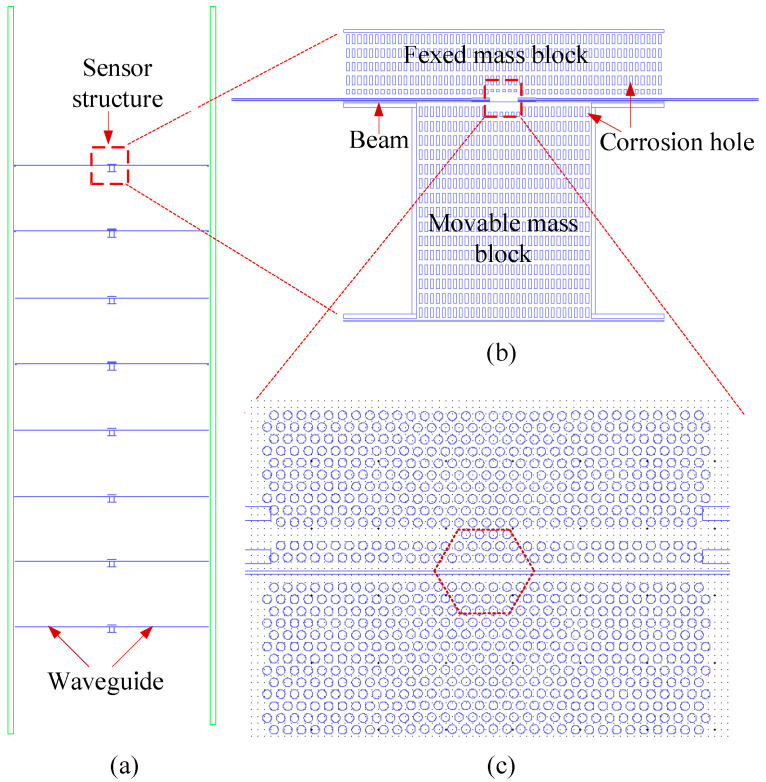
Sensor chip layout. (**a**) Overall architecture of the sensor chip; (**b**) Micromechanical resonator structure; (**c**) Photonic crystal cavity structure.

Figure 1 shows the layout of the sensor chip. As illustrated in Figure 1a, the overall chip structure measures approximately 1.5 cm × 0.5 cm and incorporates eight sets of core sensor units. Each unit primarily consists of a micromechanical resonator (Figure 1b) and a photonic crystal microcavity (Figure 1c). The photonic crystal microcavity is formed by two suspended photonic crystal slabs separated by a 100 nm air slot. The hexagonal region shown in Figure 1c corresponds to a defect-based nanocavity created by shifting several air holes. A coupling structure and a photonic crystal waveguide are formed in the upper slab by selectively removing part of the third row of air holes, enabling optical coupling between the conventional silicon waveguides and the photonic crystal waveguide, as well as between the photonic crystal waveguide and the microcavity.

The movable mass block measures 120 μm × 150 μm, and the fixed mass block has dimensions of 220 μm × 50 μm. The photonic crystal region occupies an area of approximately 16 μm × 12 μm.

The chip is fabricated on a silicon-on-insulator (SOI) substrate with a 250 nm thick top silicon layer, a 3 μm thick buried oxide layer, and a 700 μm thick handle silicon substrate. The silicon waveguides, which have a width of 0.47 μm in the main region, are integrated on both sides of the photonic crystal area and extend to the edge of the chip.

During operation of a cavity optomechanical sensor, input laser light—typically near the 1550 nm telecommunications wavelength—is evanescently coupled from the left-side silicon waveguide into a photonic crystal waveguide, followed by resonant excitation of the photonic crystal nanocavity mode. Within the nanocavity, the circulating optical field is strongly enhanced, giving rise to enhanced optomechanical coupling between the confined light and the co-localized micromechanical resonator via radiation pressure or optical gradient forces. The output optical signal from the right-side waveguide encodes information from this optomechanical interaction, manifesting as changes in the resonance frequency, amplitude, and phase of the transmitted or reflected light. High-precision measurement of these changes enables the detection of minute physical quantities, including nanoscale displacements, piconewton-level forces, and inertial accelerations.

### 2.2. Process Flow Design for MgF_2_-Protected Vapor-Phase Release

**Figure 2:**
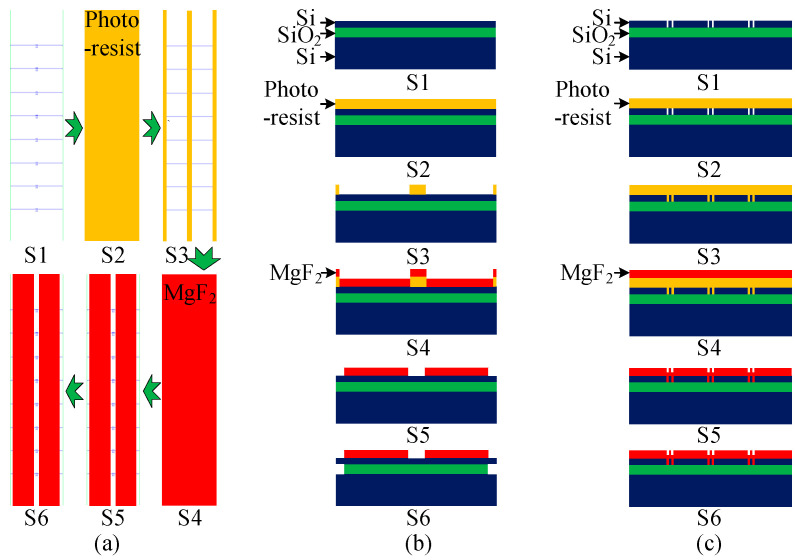
Schematic diagram of the MgF_2_-based protective release process (**a**) top-view, (**b**) Front view, (**c**) Side view.

The following section details the complete microfabrication process flow developed to integrate the MgF_2_ protection layer for vapor-phase HF release. The sequence of key steps, from substrate preparation to final release, is schematically illustrated in Figure 2.

**S1: Chip etching processing**

Photolithography and inductively coupled plasma (ICP) etching were employed to define the micro-opto-electro-mechanical system (MOEMS) structures and integrated waveguides in the device layer of SOI. The specific process proceeded as follows:

(1) RCA cleaning was performed to remove organic and ionic contaminants.

(2) A 250 nm layer of electron-beam resist (ZEP 520) was spin-coated at 3000 rpm for 45 s.

(3) Edge bead removal was conducted using ultraviolet edge exposure followed by post-application baking at 180 °C for 90 s.

(4) Patterns were exposed via electron-beam lithography (JEOL JBX-9500FS, 100 kV acceleration voltage, 2 n A beam current), developed in ZEDN50 solution for 60 s, and rinsed in IPA.

(5) ICP etching was performed using a C_4_F_8_/SF_6_ gas mixture (120/40 sccm, 10 mTorr, 800 W coil power, 20 W platen power) to achieve highly anisotropic silicon etching.

(6) The wafer was diced into 15 mm × 5 mm chip units using a laser dicing system (DISCO DFL7340, 1064 nm wavelength) along the crystal orientation. A multi-pass laser ablation process was employed to create 30 μm wide kerfs, with optimized laser parameters (2.5 W power, 50 kHz pulse frequency, and 80 mm/s scanning speed) to effectively control the heat-affected zone and prevent damage to microstructures.

(7) The edges of the diced chip units were precision-finished using chemical mechanical polishing (CMP): Diamond-based slurry (0.5 μm particle size) was applied at 5 psi pressure to polish the cut surfaces, eliminating microcracks and surface defects induced by laser processing. This process achieved an edge roughness (Ra) better than 0.1 μm, meeting both optical edge coupling requirements and mechanical reliability standards.

**S2: Photoresist coating**

Residual photoresist from previous processing steps was removed via immersion in acetone at 60 °C for approximately 2 h. In cases of denatured photoresist that proved difficult to remove, the chip samples were treated in N-Methyl-2-pyrrolidone (NMP) at 80 °C for 1 h to ensure complete stripping of resist residues. Following this cleaning process, a positive-tone photoresist was spin-coated onto the chip surface at 2000 rpm for 40 s, yielding a uniform film with a thickness of approximately 5 μm–6 μm.

**S3: Photoresist Development**

Photolithography was performed using a mask aligner (Karl Suss MA6, 365 nm wavelength, 20 mW/cm^2^ intensity, 20 s exposure) to expose and develop the photoresist. This process defined the protective pattern by removing photoresist from the waveguide regions, thereby exposing the underlying SiO_2_ in these areas for the subsequent deposition of the MgF_2_ protective layer. Conversely, the photoresist was intentionally preserved over the central MOEMS structures and over a 10–15 μm wide peripheral region along the chip edge. This reserved photoresist acts not as a protector for the MOEMS structures themselves, but as a sacrificial layer to prevent MgF_2_ deposition in these regions, thereby ensuring they remain accessible for the subsequent HF release etch and that the waveguide end-facets are cleanly exposed after liftoff.

**S4: MgF_2_ Deposition via Precision Optical Coating**

A 500 ± 15 nm thick MgF_2_ layer was deposited using an optical coating system (OTFC-900) under the following process conditions:

(1) Working vacuum: 10^−3^ Pa

(2) Substrate temperature: 80 °C ± 2 °C. Although higher temperatures (250 –300 °C) yield MgF_2_ films with superior density and stress characteristics, such elevated temperatures can induce lateral displacement of the chip’s waveguide structures. Therefore, 80 °C was selected based on experimental evaluations and is consistent with the low-temperature approach recommended in the literature to minimize tensile stress and prevent microcracking in MgF_2_ films, thereby preserving the integrity of delicate microstructures [17].

(3) Deposition rate: 0.08 nm/s (controlled via the HOM2-R-VIS350A optical thickness monitor).

(4) Planetary rotation: 15 rpm (ensuring film thickness uniformity within ±2%).

(5) Owing to the low-temperature deposition, a 10 cm radio-frequency ion source (OIS-Four) was employed to enhance layer density and uniformity.

The MgF_2_ film conformally coated the waveguide sidewalls and trenches, forming an effective diffusion barrier against HF vapor corrosion. Optical characterization confirmed that due to MgF_2_’s low refractive index (*n* = 1.38), the introduced transmission loss was negligible (<0.1 dB/cm at 1550 nm wavelength).

**S5: Selective MgF_2_/Photoresist Liftoff**

Liftoff was performed by immersing the chip in acetone at 50 °C for 12 h to remove the photoresist and the overlying MgF_2_ from the central MOEMS structures and the peripheral regions, as defined in Step S3. This critical step exposed the underlying SiO_2_ in these areas, allowing for their subsequent release by HF vapor, while simultaneously revealing the waveguide optical facets for light coupling. Importantly, the MgF_2_ layer protecting the SiO_2_ beneath the waveguide regions remained intact, thereby preserving the support structures essential for preventing waveguide collapse during the final release etch.

**S6: Vapor-Phase Release Etch**

Anhydrous HF etching (40 °C, 99 Torr, 200 nm/min, 1–2 min) was performed through etch holes in MOEMS structures. The MgF2 barrier effectively prevented HF penetration into waveguide-adjacent regions, preserving >95% of the underlying BOX layer while allowing complete release of mechanical structures.

**Figure 3:**
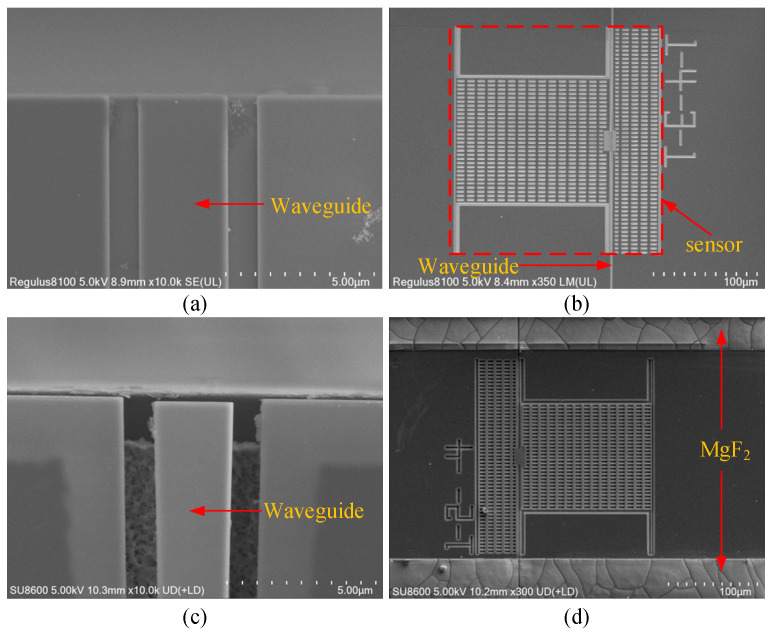
Fabrication status of the sensor: (**a**,**b**) after Step S1, (**c**,**d**) after Step S1–S6.

Following the completion of the entire fabrication process (Steps S1–S6), the resulting structural characteristics are shown in Figure 3. As illustrated in the SEM images in Figure 3a,b, the waveguide facets fabricated in Step S1 exhibit smooth morphology, and the core sensor structures are accurately defined with high etching completeness. Figure 3c,d, corresponding to the post-processed chip after Steps S2–S6, clearly show that the MgF_2_-protected silicon waveguides maintain nearly intact buried oxide (BOX) supporting layers, effectively preventing failure modes such as waveguide collapse or buckling. Furthermore, the waveguide end-facets at the chip edge remain clearly exposed, which is highly beneficial for efficient end-fire coupling with lensed fibers. In contrast, unprotected regions are completely undercut by hydrofluoric acid, resulting in full release of the movable sensor core structures. These results conclusively demonstrate the essential role of the MgF_2_ protection layer in preserving structural integrity during vapor-phase HF release. Control experiments unequivocally confirmed that the MgF_2_ protection layer is indispensable for maintaining the integrity of the long access waveguides (~5000 µm); its absence resulted in the catastrophic collapse of these waveguides, which severed the optical path to the microcavity and thus precluded the excitation of optical resonances and any meaningful device characterization.

## 3. Test and Validation

**Figure 4:**
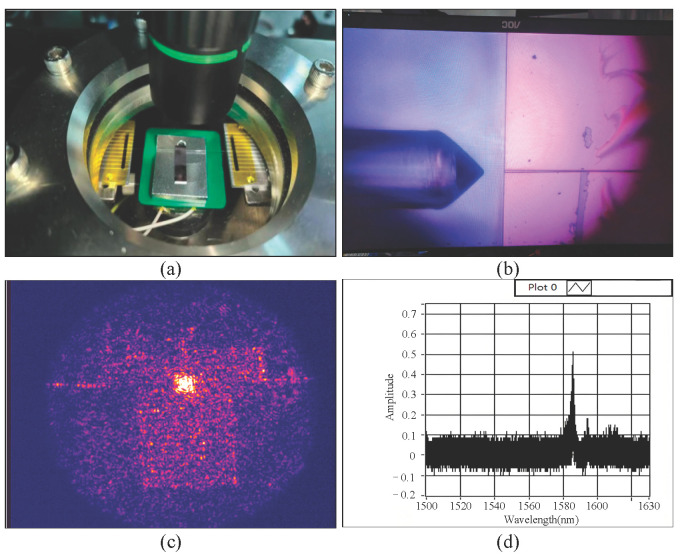
Testing of the cavity optomechanical accelerometer: (**a**) Accelerometer chip and vacuum chamber testing system; (**b**) Edge coupling between lensed fiber and waveguide; (**c**) Microcavity resonance signal observed under an infrared CCD; (**d**) Optical spectrum acquired in LabVIEW.

To evaluate the performance and structural integrity of the fabricated device, the cavity optomechanical sensor chip was tested in a high-vacuum measurement chamber using the setup shown in Figure 4a. The system consists of a laser source, polarization controller, photodetector, and other essential components. Input laser light was end-fire coupled from the left-side lensed fiber into the silicon waveguide, then evanescently coupled via the photonic crystal waveguide into the photonic crystal nanocavity. The output signal, modulated by the optomechanical interaction in the sensor, was collected by a corresponding lensed fiber on the right side, converted by a high-speed photodetector, and acquired and processed by a LabVIEW 2023-controlled data acquisition system.

In situ monitoring of light propagation and cavity mode excitation was achieved using aligned visible and infrared CCD cameras (Figure 4b,c). The transmission spectrum of the device was recorded to identify optical resonance peaks and quantify optical losses (Figure 4d). Furthermore, target physical quantities and their variations can be extracted by demodulating optomechanically induced shifts in resonance frequency, amplitude variations, or phase changes within the optical spectrum.

As shown in Figure 4c, a well-confined infrared spot is observed within the photonic crystal nanocavity region, with clearly guided propagation toward the output waveguide. Correspondingly, the transmission spectrum in Figure 4d exhibits well-defined resonance peaks, from which a primary dip at 1585.7 nm with a full width at half maximum (FWHM) of 1.75 nm is characterized, yielding a quality factor (Q) of approximately 906.

The presence of these optical signatures—both the spatially resolved guided mode and the spectrally distinct resonance—provides definitive evidence that the MgF_2_ protection scheme successfully maintained the structural and optical integrity of the suspended access waveguides. It is noteworthy that the total optical path incorporates waveguides spanning ~5000 µm in length, with each side of the chip requiring a ~2500 µm long suspended section to meet chip-level fabrication constraints, particularly to ensure mechanical robustness during processing steps such as edge polishing. The fact that such extensive suspended waveguide networks survived the release process without collapse underscores the efficacy of our method in overcoming this central fabrication challenge.

While the measured Q factor is moderate and largely reflects the current photonic crystal cavity design—which was not optimized for ultra-high Q performance—it unambiguously verifies the functional viability of the overall fabrication process. Taken together, these results confirm the successful implementation of the MgF_2_-assisted release technique and its critical role in preserving integrated silicon waveguides during vapor-phase HF etching.

The successful optical functionality demonstrated above hinges on the unique material strategy employed during fabrication. The choice of MgF_2_ as the protective layer was deliberate, following an evaluation of alternative materials. Polymer-based schemes, such as thick photoresists or flexible films, were found to be unsuitable due to their inability to form a reliable hermetic seal against HF vapor penetration. Conversely, other hard dielectric films (e.g., Si_3_N_4_, Al_2_O_3_), while offering greater resistance than SiO_2_, still exhibit a finite etch rate in HF and—most critically—would become a permanent part of the device, potentially altering the optical mode properties and precluding the realization of an all-silicon/silica platform.

MgF_2_ uniquely addresses these challenges by combining near-perfect HF inertness with a decisive processing advantage: it can be precisely patterned and selectively removed via a standard liftoff process. This allows it to permanently protect the critical waveguide regions while being cleanly stripped from the MOEMS and peripheral areas, facilitating their release and the exposure of optical facets. Moreover, an additional optical benefit is realized: the refractive index of MgF_2_ (~1.38) closely matches that of the SiO_2_ cladding (~1.45). By creating a more symmetric optical environment around the waveguide core than an air cladding, the permanent MgF_2_ cap helps reduce scattering losses and enhances transmission efficiency. This unique combination of absolute protection, standard process compatibility, selective integration, and inherent optical enhancement underscores the novelty and practicality of the presented approach.

## 4. Conclusions and Discussion

This study proposes and experimentally validates a fabrication process utilizing a magnesium fluoride (MgF_2_) protective layer, effectively resolving the critical challenge of waveguide collapse during HF vapor etching of SOI cavity opto-mechanical sensors. The core innovation lies in leveraging MgF_2_’s high chemical inertness and extremely low etch rate in HF vapor to shield the underlying silicon dioxide (SiO_2_) layer from lateral erosion, thereby maintaining structural integrity and preventing collapse.

The process flow encompasses MgF_2_ deposition, patterning, and subsequent removal steps, fully compatible with standard micro/nanofabrication techniques. Experimental results confirm successful release of silicon waveguides up to 5000 μm in length. Optical characterization reveals distinct resonance peaks and low transmission loss, verifying optical path integrity and minimal impact of the MgF_2_ layer on optical performance.

Despite these advances, several practical challenges persist, including MgF_2_ film cracking, edge delamination during polishing, and photoresist residue. These issues primarily stem from process immaturity and can be mitigated by optimizing etching parameters, deposition conditions, and process sequencing, highlighting the critical importance of precise control during thin-film processing and stripping steps.

This study not only provides a reliable fabrication strategy for high-performance integrated opto-mechanical sensors but also highlights the application potential of selective inert material base protection schemes in MEMS–photonics integration. Future work will focus on: further optimizing MgF_2_ deposition uniformity and adhesion, extending this method to more complex multilayer structures, and evaluating device long-term reliability under actual operating conditions.

Specifically, a thorough investigation into the long-term mechanical reliability of the MgF_2_ layer under practical operating conditions is warranted. Although the low-temperature, ion-assisted deposition strategy was implemented to mitigate film stress and enhance adhesion [17,18,19], quantitative assessments of adhesion strength (e.g., via scratch test), resistance to crack propagation, and performance stability under prolonged mechanical fatigue or thermal cycling remain essential future work. Establishing these reliability metrics will be a critical step in transitioning this promising fabrication technique from laboratory validation to field-deployable sensor systems.

The core contribution of this work is a manufacturing innovation that fundamentally addresses the yield-limiting challenge of waveguide collapse. It is important to note that the measured device performance demonstrates the successful fabrication of a functional sensor. However, the ultimate performance limits, such as the maximum Q factor or minimum noise floor, are predominantly set by the device’s architectural design (e.g., optical cavity and mechanical resonator design). Therefore, this process should be recognized as a critical enabling technology that provides a reliable fabrication path for a broad class of optomechanical sensors, upon which future designs can be built to push the limits of specific performance metrics.

Finally, looking beyond device-level performance, the scalability of this process to wafer-level manufacturing presents another set of challenges. To achieve high yield on a wafer scale, several engineering obstacles must be overcome. First, achieving uniform MgF_2_ film thickness and controlled stress across a full wafer requires precise optimization of deposition parameters and substrate rotation. Second, high-throughput patterning of the protective layer necessitates advanced lithography tools (e.g., steppers), which increases process cost. Third, ensuring uniform HF vapor exposure and microstructure release over large areas demands careful reactor design and process control to prevent area-dependent undercutting rates. Finally, effective wafer-level cleaning after the vapor-phase release to remove residues without causing stiction or damage to the extensive array of released microstructures becomes critically important. Addressing these challenges in thin-film engineering and batch-processing techniques is an essential next step for transitioning this promising fabrication method from laboratory validation to industrial adoption.

## Data Availability

The original contributions presented in this study are included in the article. Further inquiries can be directed to the corresponding author..
